# OnabotulinumtoxinA for treatment of chronic migraine: PREEMPT 24-week pooled subgroup analysis of patients without medication overuse

**DOI:** 10.1186/1129-2377-1-S14-P204

**Published:** 2013-02-21

**Authors:** HC Diener, DW Dodick, RE DeGryse, CC Turkel

**Affiliations:** 1 University of Essen, Dept. of Neurology, Essen, Germany; 2 Mayo Clinic Arizona, Dept. of Neurology, Phoenix, AZ, USA; 3 Allergan, Irvine, CA, USA

## Introduction

CM is a prevalent, disabling primary headache disorder. Most patients in CM clinical trials overuse AHM. The efficacy of prophylactic medications in CM patients without overuse of AHM is unclear.

## Objective

To evaluate the efficacy and tolerability of onabotulinumtoxinA in a chronic migraine (CM) subgroup without acute headache medication (AHM) overuse (MO-No).

## Design/methods

PREEMPT (two phase 3 studies: 24-week, double-blind, placebo-controlled, parallel-group phase, followed by 32-week, open-label phase) evaluated onabotulinumtoxinA for prophylaxis of headaches in CM (¡Ý15 days/month with headache lasting ¡Ý4 hours/day). Patients were stratified based on AHM use during 28-day baseline and randomized (1:1) to onabotulinumtoxinA (155-195U) or placebo every 12 weeks. Multiple headache-symptom measures were evaluated at Week 24, including mean change from baseline in headache-day frequency (primary). Pooled results from MO-No subgroup are reported.

## Results

480 (n=243 onabotulinumtoxinA; n=237 placebo) of 1384 patients met MO-No criteria. At Week 24, onabotulinumtoxinA treatment significantly reduced headache-day frequency compared to placebo (-8.8/onabotulinumtoxinA; -7.3/placebo: p=0.013). Significant improvements from baseline (p¡Ü0.027) also favored onabotulinumtoxinA at Week 24 for frequency of migraine-days, moderate/severe headache-days, total cumulative hours of headache on headache-days, and percent of patients with severe (¡Ý60) headache impact test (HIT-6) scores. Improvements in total HIT-6 and migraine-specific questionnaire scores all significantly favored onabotulinumtoxinA over placebo at Week 24 (p¡Ü0.032). Few patients in this subgroup discontinued because of an adverse event (AE); AEs were consistent with overall PREEMPT tolerability.

## Conclusion/relevance

OnabotulinumtoxinA is effective and well-tolerated for prophylaxis of headache in CM patients who do not overuse AHM.

## Support

Allergan, Inc.

